# Field epidemiology capacity of the national veterinary services of Lao PDR: An online survey

**DOI:** 10.3389/fvets.2023.1096554

**Published:** 2023-03-21

**Authors:** Supatsak Subharat, Dethaloun Meunsene, Vannaphone Putthana, Harish Tiwari, Simon M. Firestone

**Affiliations:** ^1^EpiCentre, School of Veterinary Science, Massey University, Palmerston North, New Zealand; ^2^Department of Veterinary Medicine, Faculty of Agriculture, National University of Laos, Vientiane, Laos; ^3^Sydney School of Veterinary Science, University of Sydney, Camden, NSW, Australia; ^4^Faculty of Science, Melbourne Veterinary School, The University of Melbourne, Parkville, VIC, Australia

**Keywords:** field epidemiology, animal health, capacity, survey, Lao PDR, Southeast Asia

## Abstract

**Introduction:**

Capacity in veterinary epidemiology is critical to detect, respond and control infectious diseases. Laos veterinary service is limited by having only a small number of veterinarians who graduated from overseas. Animal science graduates support the majority of the Laos veterinary services. The veterinary program was established in 2009 at the National University of Laos. We aimed to understand the national veterinary epidemiology capacity and identify gaps and training needs.

**Method:**

A cross-sectional online study was conducted in 2021 targeting central (DLF), provincial (PAFO) and district (DAFO) government animal health officers and veterinary/animal science academics (*n* = 332). The questionnaire addressed skills, experiences and perceived training needs in outbreak investigation, disease surveillance, data management and analysis, epidemiological surveys, One Health, leadership and communication and biosecurity. A descriptive analysis was performed and associations between demographic factors and epidemiological skills were examined.

**Results and discussion:**

In total, 205 respondents completed the questionnaire (61.8% response rate). Respondents reported low or no skills and experience in data management and analysis, epidemiological surveys and One Health. In contrast, higher but limited skills and experiences were reported in outbreak investigation, disease surveillance and biosecurity. Previous epidemiology training was primarily associated with stronger experiences in veterinary epidemiology competencies, followed by respondents that had completed a veterinary degree, highlighting the value of the currently available epidemiology training and veterinary-trained personnel in Lao PDR. This study could help inform the Laos government in its policy planning for field veterinary epidemiology capacity and future training.

## Introduction

Veterinary epidemiology deals with studying diseases and health events in animal populations. It has various useful tools for animal disease investigation and control by identification of trends and patterns in the occurrence of diseases in populations and for conducting risk assessments (1). These skills are considered essential for the animal health workforce to prevent, detect and control infectious diseases. However, it has been recognized that veterinary epidemiology skills are in short supply even in developed countries. The needs and gaps are more severe in developing countries such as the Lao People's Democratic Republic (PDR), where there are relatively few qualified veterinarians.

Lao PDR currently has a human population of 7.1 million, with a gross domestic product (GDP) of USD 17.95 billion (2). The agricultural sector contributes 20% of the GDP, where rice production is a primary activity (3). Smallholder livestock farm systems (i.e., cattle, buffaloes, pigs and goats) account for 18% of Lao's agriculture GDP (3). The animal health workforce of Lao PDR is predominantly employed by the Department of Livestock and Fisheries (DLF) under the Ministry of Agriculture and Forestry (MAFF). DLF is responsible for meeting Lao PDR's requirements for animal disease surveillance as a member country of the World Organization for Animal Health (WOAH) and the South-East Asia and China Foot and Mouth Disease (SEACFMD) control program (4). Lao PDR is considered a major thoroughfare for animal trade between neighboring countries and China (5, 6), also with extensive local movements (7), both of critical importance for the regional spread of major livestock diseases. Livestock production in Lao PDR is impacted by various endemic diseases (foot and mouth disease and hemorrhagic septicemia) and emerging infectious diseases (African swine fever, lumpy skin disease, highly pathogenic avian influenza), which cause significant economic losses to the livestock industries as well as impacting food security and public health (8–11).

Field veterinary epidemiology skills are important to support animal disease prevention and control in Lao PDR. A strong national veterinary epidemiology capacity will enhance the effectiveness of surveillance systems and improve disease reporting and responses to outbreaks. Currently, the Laos animal health services are mainly supported by animal science graduates as the number of qualified veterinarians is limited. Before the National University's Faculty of Agriculture established a veterinary faculty in 2009 and produced its first veterinary graduates in 2014, veterinary education was unavailable in Lao PDR, with a very limited number of local veterinarians having been trained in Thailand and other countries. In addition, resource gaps remain related to recruiting sufficient numbers of newly graduated veterinarians to the public service (12). Lao PDR veterinarians have opportunities for advanced training in veterinary epidemiology in the region, i.e., Field Epidemiology Training Programs for Veterinarians (FETPVs) conducted in Thailand (13) and *ad hoc* in-service training supported by international donors such as FAO, WOAH, Australian and New Zealand governments (5, 14–16). Nevertheless, these opportunities are few for the animal health workforce to continue strengthening their capabilities.

This study aimed to describe the field veterinary epidemiology capacity in Lao PDR based on the FAO technical guidelines (1) and identify training needs in veterinary epidemiology to better inform the national animal health workforce development strategy. More specifically, the intention was to identify any knowledge gaps that could inform the development of online veterinary epidemiology training modules by the Asia Pacific Consortium of Veterinary Epidemiology (APCOVE) to strengthen the veterinary workforce in Asia-Pacific, including Lao PDR for infectious disease detection and response.

## Materials and methods

### Study design

A cross-sectional online study was conducted in Lao PDR between February and March 2021. The target population was the animal health workforce, including government veterinarians at the national level (DLF), provincial-level agricultural and forestry officers (PAFO), district-level agricultural and forestry officers (DAFO), and academics from Laos university. Assuming the population size of the animal health workforce in Lao PDR to be 500 and that 50% of them use epidemiologic skills, the study required a sample size of 218 to estimate the proportion of veterinarians using epidemiologic skills with 5% absolute precision and 95% confidence. The sample size was calculated using Statulator (17).

### Online questionnaire survey

A questionnaire was developed in English, translated to Laos and piloted to test for comprehension and appropriateness by local staff before data collection (Supplementary material). The questionnaire comprised 30 questions seeking information about epidemiological-related experiences to seven competencies that respondents had performed in the past 3 years. Competencies included outbreak investigation, animal disease surveillance, data management and analysis, epidemiological surveys and studies, One Health (defined as “a collaborative, multisectoral, and trans-disciplinary approach with the goal of achieving optimal health outcomes recognizing the interconnection between people, animals, plants, and their shared environment”), leadership and communication and biosecurity. The questionnaire also asked for their opinions on future training priorities and their demographic characteristics (i.e., age, work position, location, degree and previous epidemiology training). Questions were a combination of close-ended and open-ended, with room for additional comments following closed questions. A sampling frame was established by the DLF veterinary services records. An invitation containing a web link to the questionnaire was sent by email and/or mobile phone messenger application to 332 identified animal health workforce personnel with follow-up reminders from DLF veterinary services. Answering the questionnaire was taken as consent. The online questionnaire was conducted using the REDCap system approved and held by the University of Sydney (18). The questionnaire was approved by the University of Sydney Human Ethics Committee (2020/459, dated 15 September 2020).

### Data

The questionnaire data were exported from the REDCap online database into a Microsoft Excel spreadsheet. Then, the data were checked for obvious errors, cleaned and recoded by the authors as appropriate prior to analysis. Briefly, the data included a unique questionnaire identifier, frequency of respondent's experience with specific activities during the last year (never, rarely, once a month and more than once a month) regarding outbreak investigation, animal disease surveillance, data management and analysis, epidemiological surveys and studies, One Health, leadership and communication, biosecurity, number of times respondents involved in such activities overall in the past 3 years and their opinion for future training priority. The demographic data included demographic characteristics of respondents (age, sex, work position, degree, and experience of previous epidemiology training).

### Statistical analysis

Descriptive analyses were performed, then prior to analytical analyses, the following variables were recategorized: epidemiology skill outcomes into three ordinal levels, i.e., never, rarely and regularly (combination of once a month and more than once a month), age into three groups (18–34 years, 35–44 years and 45–64 years), prior study into a binary variable (veterinary degree vs. non-veterinary degree), and experience of previous epidemiology training into binary variables (yes vs. no) per specific activity. Next, the associations between each epidemiology skill outcome and potential explanatory variables, including demographic variables, were tested using Chi-squared or Fisher's exact tests, as appropriate depending on sample sizes. Then, these associations were further assessed using ordinal logistic regression models to adjust for age, sex and prior epidemiology training experience. As the respondents' positions and degrees were highly correlated, regression models were built separately for these two variables. Data processing and analyses were carried out using R version 4.2.1 (19). The ordinal logistic regression (proportional odds) models were built using R package *MASS* (20) and tested for parallel regression assumption using R package *brant* (21). Outputs are reported as estimated odds ratios for variables associated with an increased likelihood of being associated with higher category levels, along with their 95% confidence intervals.

## Results

### Respondents and their demographics

There were 205/332 (62.8%) respondents who completed the online questionnaire. The demographics of respondents are shown in Table 1. Half of the respondents identified as DAFO, 13% as PAFO, 6% as DLF officer, 11% identified as others, e.g., academics and 20% not specified. The majority of respondents (56%) were aged between 35 and 44 years old, 27% were aged between 25 and 34 years old, 10% were aged between 45 and 54 years old, 1% were aged between 55 and 64 years old and 5% did not specify. The majority of respondents were male (73%). For degree, 51% had completed an Animal Science degree (34% Bachelor, 15% diploma, and 2%), whereas 9% had completed a Veterinary Science degree (4% Bachelor, 4% Masters and 1% PhD). Forty per cent had other educational backgrounds (15%) or did not specify (25%). Only 47% had received some form of prior epidemiology training, i.e., workshop, FETPV, Post-graduate training and others.

**Table 1:** Demographic characteristics of the Lao PDR animal health workforce respondents participating in the field veterinary epidemiology capacity online cross-sectional study between February and March 2021 (*n* = 205).

**Characteristics**	**Number**	**Percentage (%)**
**Age (years)**		
18–24	2	1
25–34	56	27
35–44	114	56
45–54	21	10
55–64	2	1
NA	10	5
**Gender**		
Male	150	73
Female	47	23
Others	4	2
NA	4	2
**Position**		
DLF	12	6
PAFO	26	13
DAFO	102	50
Others, e.g., academics	23	11
NA	42	20
**Degree**		
Diploma in Animal Science	30	15
Bachelor in Animal Science	70	34
Master in Animal Science	4	2
Bachelor in Veterinary Science	8	4
Master in Veterinary Science	8	4
PhD	2	1
Others	31	15
NA	52	25
**Previous epidemiology training (not mutually exclusive)**		
None	109	53
Epidemiology workshop	80	39
FETPV	33	16
Post-graduate training	2	1
Others	22	11

### Outbreak investigation

**Figure 1:**
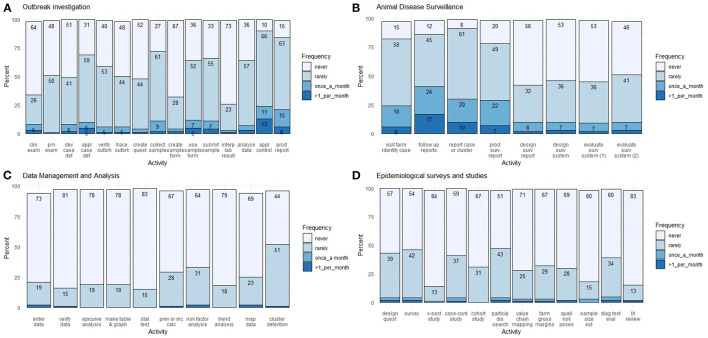
Percentage frequency distribution of respondent's activity regarding outbreak investigation **(A)**, animal disease surveillance **(B)**, data management and analysis **(C)** and epidemiological surveys and studies **(D)** from an online veterinary epidemiology capacity cross-sectional study in Lao PDR between February and March 2021. The percentage may not sum to 100 due to rounding. Key labels on the x-axis: clin, clinical; pm, post-mortem; def, definition; quest, questionnaire; interp, interpret; surv, surveillance; prev, prevalence; inc, incidence; calc, calculation; X-sect, cross-sectional; particip, participatory; Quali, Qualitative; asses, assessment; est, estimation; diag, diagnostic; eval, evaluation; lit, literature.

The breakdown of responses related to outbreak investigation skills is presented in Figure 1A. In general, most respondents never or rarely applied these skills. For example, the majority of respondents had no experience in conducting clinical examination for case detection and diagnosis (64% never), conducting post-mortem examination for case detection and diagnosis (48% never), developing case definitions to classify animals or farms into cases and non-cases (51% never), verifying that an outbreak is occurred (40% never), conducting trace-forward and trace-backward searches to identify other cases (48% never), creating an outbreak investigation questionnaire (52% never), creating sample submission forms (67% never), interpreting laboratory results (73% never), analyzing data from an outbreak by animal/space/time (36% never).

Fewer participants reported no experience in other aspects of outbreak investigation, such as applying case definitions to classify animals or farms into cases and non-cases (31% never), collecting samples (27% never), utilizing sample submission forms (36% never), transporting samples to the laboratory (33% never), applying preliminary control strategies to contain the outbreak (10% never) and producing an outbreak report (15% never).

In terms of frequency, most respondents had led or participated in an outbreak investigation in the past 3 years (17% > 6 times, 14% 3–6 times, 33% 1–2 times and 32% never). In addition, they indicated high priority for future training in this competency (38% very high, 43% high, 9% moderate and 8% low priority).

**Figure 2:**
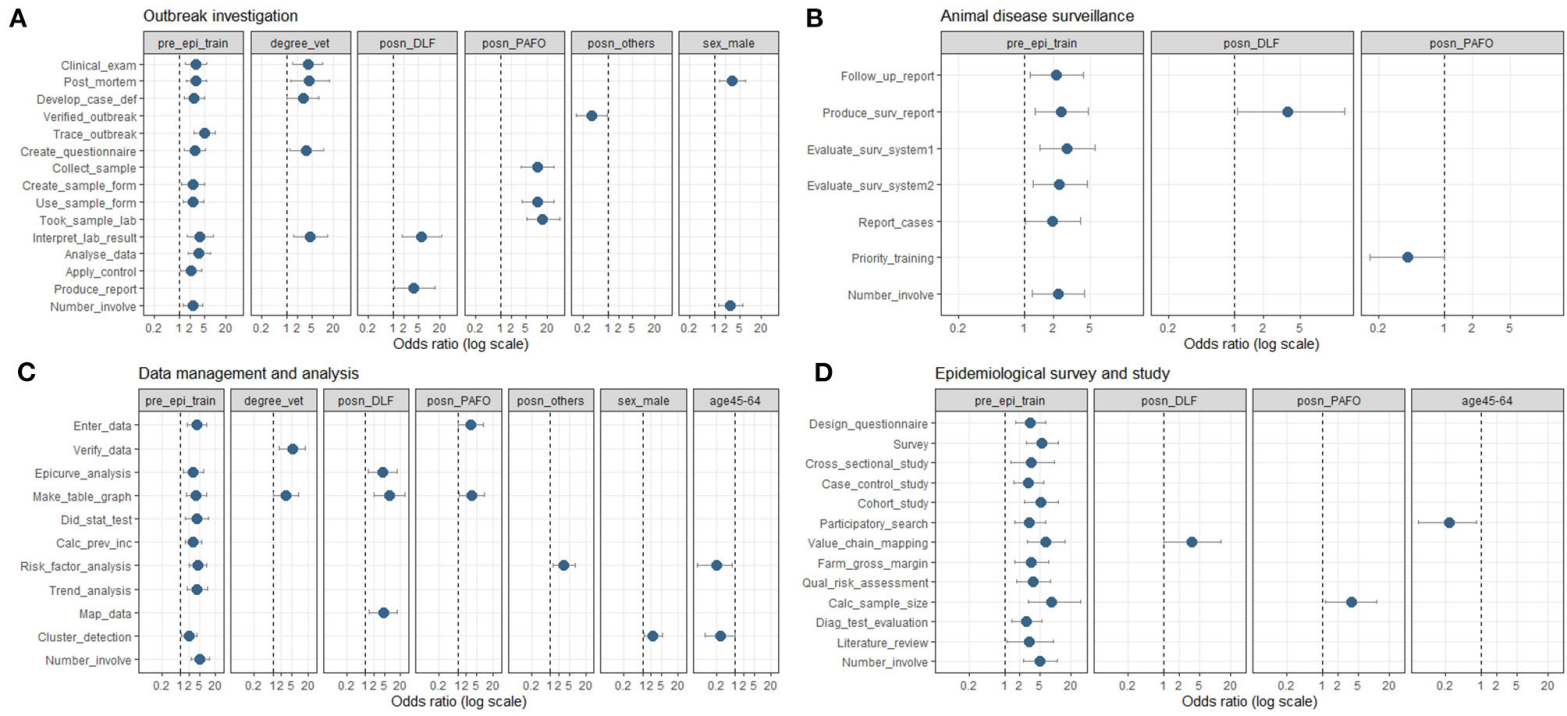
Statistical significant associations (*p* < 0.05) between skill outcomes and demographic variables for outbreak investigation **(A)**, animal disease surveillance **(B)**, data management and analysis **(C)**, and epidemiological surveys and studies **(D)**, based on multivariable ordinal logistic regression models adjusting for age, sex and prior epidemiology training experience from an online veterinary epidemiology capacity cross-sectional study in Lao PDR between February and March 2021. The point estimate of odds ratio (OR) is depicted as a circle, with the error bar indicating the 95% confidence interval. Key labels on the y-axis: def, definition; surv, surveillance; stat, statistical; calc, calculation; prev, prevalence; inc, incidence; Qual, Qualitative; Diag, Diagnostic.

The statistically significant associations (*p* < 0.05) between outbreak investigation skills and demographic variables are shown in Figure 2A. Briefly, respondents with previous epidemiology training or a veterinary degree were more likely to conduct many of the outbreak investigation activities. Compared to DAFO, the PAFO staff were more involved in field sample collection and submission, whereas the DLF staff were more involved in more advanced activities such as interpreting laboratory results and writing outbreak investigation reports. On the other hand, the academic staff were less likely to be involved in the field. In addition, the male respondents were likelier to be involved in field outbreak investigation and conduct post-mortem examinations than female respondents.

### Animal disease surveillance

The responses for animal disease surveillance skills are shown in Figure 1B. Most respondents had little experience in these skills for more advanced disease surveillance activities. For example, the majority of respondents had no experience in designing a surveillance summary report template that can be used periodically (56% never), designing a surveillance system (53% never), evaluating the operation and disease reporting components of a surveillance system (53% never) and identifying the strengths, limitations and gaps of a surveillance system (46% never).

For basic disease surveillance activities, fewer respondents had no experience in visiting farms and talking with farmers to identify possible cases (15% never), follow-up reports from informal sources (12% never), reporting cases or clusters of cases to the appropriate authorities (8% never), producing a surveillance summary report (20% never).

In terms of frequency, most respondents have led or participated in animal disease surveillance in the past 3 years (16% > 6 times, 16% 3–6 times, 37% 1–2 times and 24% never). In addition, they indicated high priority for future training in this competency (37% very high, 45% high, 9% moderate and 5% low priority).

The statistically significant associations (*p* < 0.05) between animal disease surveillance skills and demographic variables are shown in Figure 2B. Briefly, respondents with previous epidemiology training were more likely to conduct animal disease surveillance activities. Compared to DAFO, the DLF staff were more involved in writing surveillance reports. Interestingly, the PAFO staff indicated less priority in surveillance training.

### Data management and analysis

The responses for skills in data management and analysis are shown in Figure 1C. Generally, most of the respondents had no or little experience in these skills. For example, the majority of respondents had no experience in entering surveillance or outbreak data into a spreadsheet program such as MS excel (73% never), verifying surveillance data for data entry errors and typos (81% never), preparing and interpreting an epidemic curve to describe the outbreak (78% never), presenting surveillance or outbreak data using tables and graphs (78% never), conducting a statistical test of hypothesis (83% never), calculating prevalence and incidence measures from surveillance data (67% never), comparing prevalence and incidence between groups to identifying risk factors (64% never), identifying trends, patterns, and outliers in surveillance data (79% never), constructing maps from outbreak or surveillance data (69% never). Interestingly, 56% of the respondents mentioned some experience in identifying suspected cluster of disease, whereas 44% had never been involved in this activity.

In terms of frequency, a small number of respondents have led or participated in data management and analysis in the past 3 years (4% > 6 times, 8% 3–6 times, 22% 1–2 times and 61% never). They indicated high priority for future training in this competency (41% very high, 41% high, 10% moderate and 4% low priority).

The statistically significant associations (*p* < 0.05) between data management and analysis skills and demographic variables are shown in Figure 2C. The respondents with previous epidemiology training were likelier to perform most data analysis and management skills. The respondents with a veterinary degree were likelier to verify data for entry errors and present data using tables and graphs. Compared to DAFO, the DLF staff is involved more in advanced analysis, such as constructing disease mapping and epidemic curves. The PAFO staff were more involved in entering and presenting data. In addition, the academic staff were more likely to be involved in the risk factor analysis. The male respondents were more likely to identify suspected clusters of disease than females. The more senior respondents were less likely to perform some data analysis skills.

### Epidemiological surveys and studies

The responses for skills in epidemiological surveys and studies are shown in Figure 1D. Generally, most of the respondents had no or little experience in these skills. For example, the respondents had no experience in designing a questionnaire for data collection (57% never), conducting a survey (54% never), conducting a cross-sectional study (84% never), conducting a case-control study (59% never), conducting conducted a cohort study (67% never), conducting a participatory disease search (51% never), conducting a value chain mapping (71% never), conducting gross margins for a farm (67% never), conducting qualitative risk assessment (69% never), estimating sample size (80% never), evaluating a diagnostic test (60% never) and conducting a literature review (83% never).

In terms of frequency, a small number of respondents have led or participated in epidemiological surveys and studies in the past 3 years (3% > 6 times, 10% 3–6 times, 22% 1–2 times and 61% never). They indicated high priority for future training in this competency (37% very high, 45% high, 98% moderate, 5% low priority and 1% not needed).

The significant associations (*p* < 0.05) between epidemiological survey and study skills and demographic variables are shown in Figure 2D. The respondents with previous epidemiology training were likelier to perform all epidemiological study and survey skills. Compared to DAFO, the DLF staff were more involved in planning and conducting value chain mapping. The PAFO staff were likelier to perform sample size calculations. However, the senior respondents were less likely to plan or conduct participatory disease searches.

### One Health

**Figure 3:**
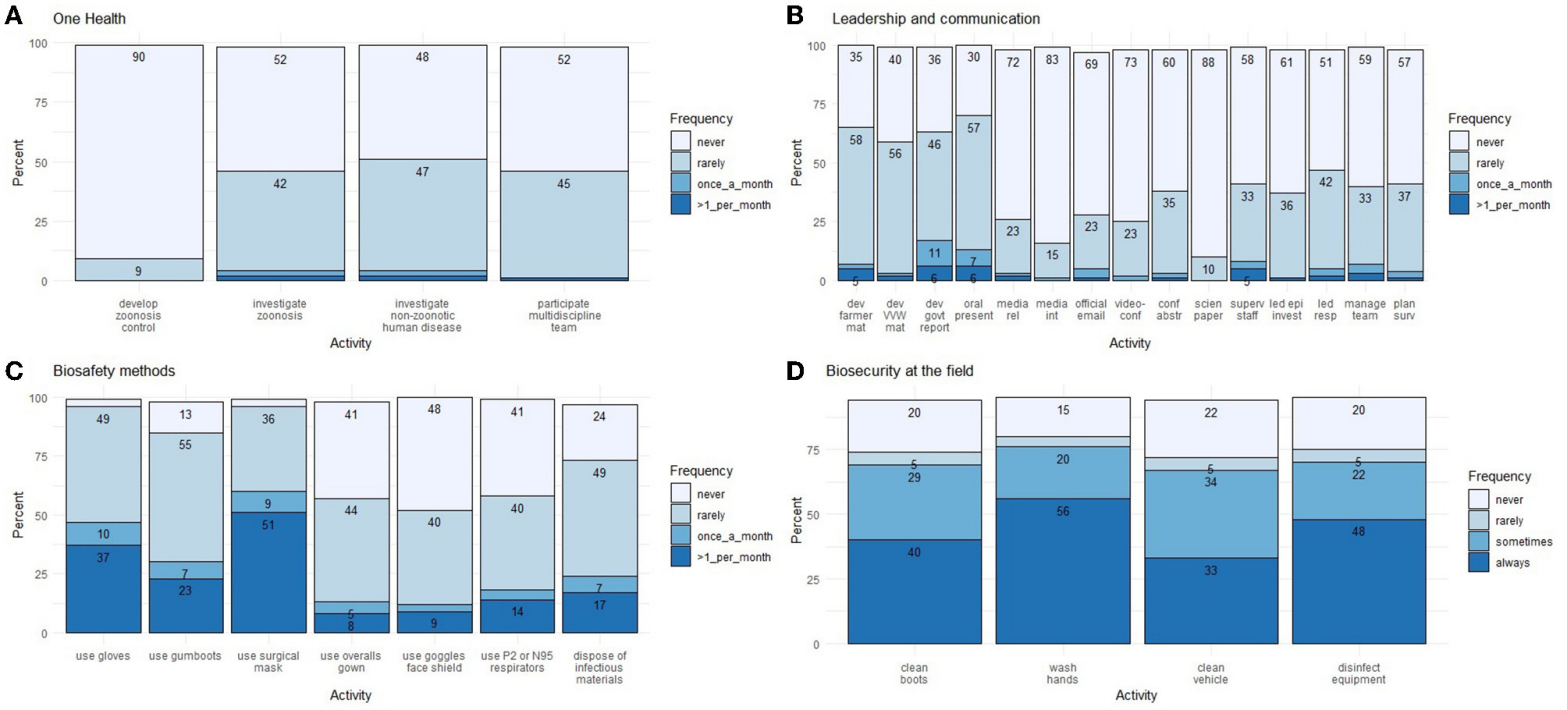
Percentage frequency distribution of respondent's activity regarding One Health **(A)**, leadership and communication **(B)**, biosafety methods **(C)** and biosecurity at the field **(D)** from an online veterinary epidemiology capacity cross-sectional study in Lao PDR between February and March 2021. The percentage may not sum to 100 due to rounding. Key labels on the x-axis: dev, develop; mat, materials; VVW, village veterinary worker; govt, government; rel, release; int, interview; conf, conference; abstr, abstract; scien, scientific; superv, supervise; epi= epidemiological; invest, investigation; resp, response; surv, surveillance.

The responses for One Health are shown in Figure 3A. Overall, the majority of respondents had no or little experience in applying One Health activities. For example, the respondents had no experience in developing zoonoses control programs (90% never), investigating zoonotic diseases (52% never), investigating non-zoonotic human diseases (48% never) and Participating in a multidisciplinary team involving professionals from animals, human, and/or environmental sectors (52% never).

In terms of frequency, a small number of respondents have led or participated in One Health in the past 3 years (3% > 6 times, 3% 3–6 times, 26% 1–2 times and 63% never). However, they indicated high priority for future training in this One Health (35% very high, 44% high, 12% moderate, 4% low priority and 1% not needed).

**Figure 4:**
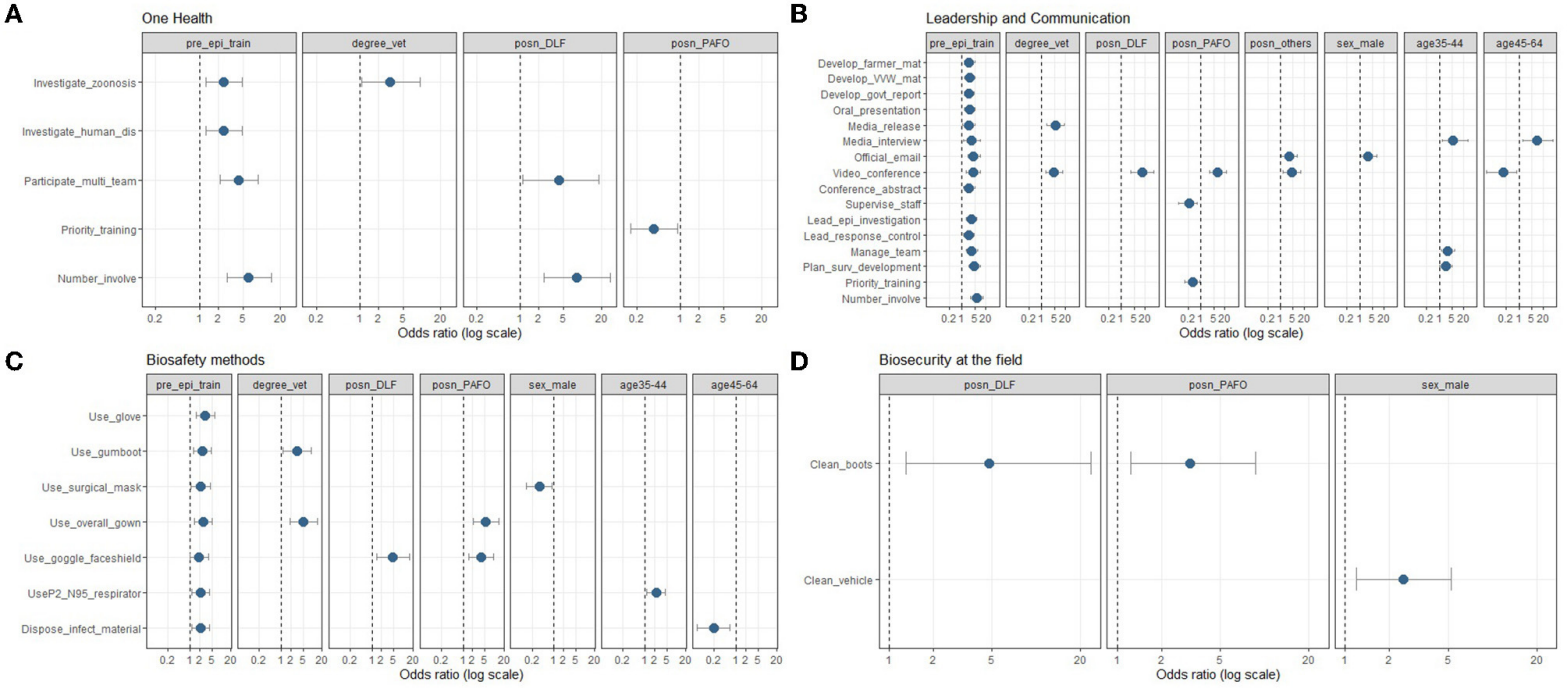
Statistical significant associations (*p* < 0.05) between skill outcomes and demographic variables for One Health **(A)**, leadership and communication **(B)**, biosafety methods **(C)** and biosecurity at the field **(D)** based on multivariable ordinal logistic regression models adjusting for age, sex and prior epidemiology training experience from an online veterinary epidemiology capacity cross-sectional study in Lao PDR between February and March 2021. The point estimate of odds ratio (OR) is depicted as a circle, with the error bar indicating the 95% confidence interval. Key labels on the y-axis: prog, program; dis, disease; mat, material; VVW, village veterinary worker; govt, government; epi, epidemiological; surv, surveillance; infect, infectious.

The significant associations (*p* < 0.05) between One Health skills and demographic variables are shown in Figure 4A. Briefly, the respondents with previous epidemiology training were more likely to work in One Health. In addition, compared to DAFO, the DLF staff were more likely to participate in a multidisciplinary team and involved in one health. Interestingly, PAFO staff were less likely to prioritize One Health training.

### Leadership and communication

The responses for leadership and communication are shown in Figure 3B. Overall, the respondents had some experience in basic communication skills but little or no experience in advanced communication skills. For example, a small proportion of respondents had no experience in developing farmer materials (35% never), developing animal health worker materials (40% never), preparing government reports (36% never), and giving an oral presentation (30% never). On the other hand, the majority of respondents had no experience in preparing a media release (72% never), giving a media interview (83% never), handling official communication by email (69% never), using video-conferencing tools (73% never), preparing an abstract for submission to a conference (60% never), and preparing a manuscript for publication in a scientific journal (88% never).

For leadership skills, the majority of respondents had no experience in supervising staff (58% never), leading an epidemiological investigation (61% never), leading a response team or a control center (51% never), Managing a team (59% never) and planning a project related to surveillance system development or implementation (57% never).

In terms of frequency, a small number of respondents have led or participated in leadership and communication in the past 3 years (4% > 6 times, 5% 3–6 times, 23% 1–2 times and 61% never). They indicated high priority for future training in these skills (35% very high, 48% high, 7% moderate, 5% low priority and 1 % not needed).

The statistically significant associations (*p* < 0.05) between leadership and communication skills and demographic variables are shown in Figure 4B. The respondents with previous epidemiology training were likelier to perform leadership and communication skills. The respondents with a veterinary degree were more likely to use video-conferencing tools and prepare a media release. Compared to DAFO, the DLF and PAFO staff were more likely to use video-conferencing tools. In addition, the PAFO staff were less likely to supervise staff and indicate a priority on leadership and communication training. The respondents in academics were more likely to use video-conferencing tools and handle official communication by email when compared to DAFO staff. The male respondents were more likely to handle official communication by email when compared to females. Compared to junior respondents (18–34 years), the respondents in mid-career (35–44 years) were more likely to plan projects, give media interviews and manage a team. The senior respondents (45–64 years) were less likely to use video conferences but more likely to give media interviews.

### Biosafety methods

The responses for biosafety methods are shown in Figure 3C. Overall, most respondents have applied biosafety measures. A small number of respondents have never used gloves (3%), gumboots (13%), or surgical masks (3%). A higher number of respondents have never used overalls gowns (41%), goggles and face shields (48%), P2 or N95 respirators (41%) and disposed of infectious materials (24%).

The statistically significant associations (*p* < 0.05) between biosafety skills and demographic variables are shown in Figure 4C. Briefly, the respondents with previous epidemiology training were more likely to apply all biosafety methods. The respondents with a veterinary degree were likelier to use an overall gown and gumboot. Compared to DAFO, the DLF staff were more likely to use safety goggles/face shields. The PAFO staff were more likely to use overall gowns and face shields. The male respondents were more likely to use a surgical mask when compared to females. Compared to junior respondents (18–34 years), the respondents in mid-career (35–44 years) were more likely to use P2 or P95 respirators. The senior respondents (45–64 years) were less likely to dispose of infectious material.

### Biosecurity measures

Most respondents have applied biosecurity measures in the field before or after visiting the farm (Figure 3D). However, a small number of respondents have never cleaned their boots (20% never), washed their hands (15% never), cleaned their vehicles (22% never) and disinfected their equipment (22% never).

The statistically significant associations (*p* < 0.05) between biosecurity measures and demographic variables are shown in Figure 4D. Compared to DAFO, the DLF and PAFO staff were more likely to clean their boots before and after visiting a farm. In addition, the male respondents were more likely to clean their vehicles when compared to females.

Most respondents indicated high priority for future training in biosafety and biosecurity methods (32% very high, 48% high, 11% moderate, 3% low priority and 2 % not needed).

## Discussion

This study described the field veterinary epidemiology capacities of the animal health workforce in Lao PDR and identified training needs in various competencies. The majority of the respondents were DAFO/animal science graduates representing the front line of the Laos veterinary service. There was no pre-existing information (e.g., by age, gender, qualification and roles and responsibilities) that allowed a comparison between respondents and non-respondents, which would have been useful for further analysis and validation.

The online questionnaire survey was chosen for this study due to its accessibility and cost-effectiveness. The drawback of this approach was the potential selection bias that may have been introduced by design, as only respondents with up-to-date contact details and access to the internet had the opportunity to participate in the study. Nevertheless, we see no reason to believe that selection bias affected the overall results presented in this study. In addition, the sample size (205 from 218 estimated) and response rate (63%) obtained were considered acceptable. The Performance Veterinary Service (PVS) pathway developed by WOAH and the Joint External Evaluation developed by the World Health Organization (WHO) are alternative tools to assess a country's capacity for animal health workforce and One Health as reported in Laos previously (12, 22).

A lack of capacity in outbreak investigation skills was identified in this study. Those who applied were limited to basic tasks and respondents described less engagement in activities that required advanced skills. The activities involved were mostly well-aligned with their work positions, e.g., PAFO were more involved in field sample collection and sample submission to the laboratory, whereas the DLF staff were more involved in high-level activities such as interpreting laboratory results and writing outbreak investigation reports. This is consistent with the known disease reporting system, where the reporting of disease outbreaks in Laos begins in the village. Farmers report unusual events to their Village Veterinary Worker (VVW), a layperson trained in basic animal health management. The VVW then report to their DAFO, which then informs PAFO and DLF central office in Vientiane, respectively (9). The DLF handles the high-level decision-making, whereas the PAFO leads field operations.

On the other hand, the academic staff with no direct responsibility reported being less involved in the field investigation. It might be useful to involve more academic staff as part of the investigation team in the future to utilize their skills in the field. In addition, direct field experience obtained could be integrated to strengthen the veterinary curriculum.

The respondents with a veterinary degree and previous epidemiology training reported higher and more advanced experience in outbreak investigation competency. Also, it is expected that male respondents were more involved in physically demanding activities in field investigation and post-mortem examination.

Limited human and financial resources often challenge animal disease surveillance in lower-middle-income countries such as Lao PDR. A recent study noted challenges for Laos' disease reporting systems within and between government sectors resulting in under-reporting disease and delayed control measures (23). Most respondents in our study had applied these skills. Still, their skills were mostly limited to basic activities such as visiting farms, following up on reports from informal sources, reporting cases, and producing surveillance reports. Additionally, among those who participated in these activities, most tasks were related to applying acquired information and not creating tools or evaluating systems.

The respondents with previous epidemiology training were more likely to conduct the surveillance activities investigated in this study. Interestingly, respondents from PAFO indicated less priority in surveillance training. This could be due to recent donor programs that PAFO has been involved in, which have already focused on various surveillance projects (23, 24) but requires further investigation to understand the reasons.

Data management and analysis are required skills for epidemiological study (25). Our results showed that the Laos animal health workforce had a significant gap in data management and analysis and skills in undertaking epidemiological surveys and studies. Most respondents had never applied these skills. Nevertheless, our study has shown that respondents from the available epidemiology training programs display increased capacity in data analysis and epidemiological studies. The gender effect observed in the identification of suspected disease clusters could be due to the field role of males in collecting data to look for disease patterns that suggest a higher than expected number of cases in a certain time period or area.

In addition, this study found that respondents from the Laos animal health workforce had little experience in One Health activities. Only respondents in central DLF or those with veterinary degrees or previous epidemiology training were involved in such activities. The Laos Joint External Evaluation (12) and PVS (22) reported that the functional mechanisms for coordinating and integrating the public health and animal health sectors had been established. Still, specific mechanisms for responding to zoonotic disease were weak, as coordination was ineffective or information sharing was not timely. The animal and human health sectors are not equally resourced, with the animal health sector lacking in terms of surveillance and laboratory systems and human and financial resources (26). Nevertheless, standard operating procedures (SOPs) for responding to specific zoonotic diseases exist in Laos, and training events and exercises have been conducted at national and sub-national levels. There are good examples of joint investigation and response to priority zoonotic diseases such as highly pathogenic avian influenza (H5N1) in the past (12).

Leadership and communication are core competencies in the field epidemiology training of veterinarians at the frontline and intermediate levels (1). Our study showed that the Laos animal health workforce has experience in basic communication skills such as developing materials, reporting and making oral presentations but little or no experience in advanced communication skills involving presenting at a scientific conference or preparing a publication. It is not unexpected that the more senior respondents would be less familiar with communication technology. Still, they would be responsible for planning projects, managing the team and giving media interviews. Interestingly, respondents with previous epidemiology training were more likely to conduct communication and leadership activities, i.e. developed education materials for farmers and VVW, prepared government report, given oral presentation, prepared a media release, given a media interview, handled official communication, utilized video-conferencing tools, prepared conference abstract, led epidemiological investigation and response, managed team and planned a surveillance development project. These indicated trainees' capability in communication and leadership. According to the FAO's guidelines, the animal health workforce should be able to communicate effectively with technical and non-technical audiences and be proficient in oral and written communication. In addition, leadership skills are required to guide and lead outbreak investigation teams and plan disease control activities (1).

Our results showed the respondents had the most experience in biosafety and biosecurity practice. Most respondents have used common protection, i.e., gloves, gumboots and surgical masks. However, it is not unexpected that a higher number of respondents have never used overalls gowns, goggles and face shields, P2 or N95 respirators due to limited availability to DLF and PAFO offices. Similarly, the respondents with previous epidemiology training were more likely to apply these skills. This highlights the biosecurity capacity of the animal health workforce due to donor control programs (27).

Overall in our study, previous epidemiology training was mostly associated with stronger experiences in veterinary epidemiology competencies, followed by respondents that had completed a degree in veterinary science. These findings highlight the value of the available epidemiology training and veterinary-trained personnel in Lao PDR. Nonetheless, it has been reported that although animal health workers participate in FETP and FETPV programs, their numbers are insufficient to meet surveillance and disease response needs, especially at provincial and district levels (12). In addition, there continues to be a critical shortage of qualified veterinarians within the Lao animal health workforce. Therefore, an option is to provide more field epidemiology training at the local level (especially DAFO), as the epidemiological capacity of the local level has been highlighted as an important way of improving the accuracy and timeliness of outbreak responses (28).

Most respondents expressed a keen interest in more training opportunities to improve their abilities across all competencies (>80% indicated high or very high priority). However, future training programs could prioritize the weakest competencies identified in this study, i.e. data management and analysis, epidemiological survey and study and One Health. Furthermore, we recommend that such training facilitate enhanced collaboration among different sectors as this process will contribute to developing a strong network. Future training should also make effective use of the existing expertise from the national level and academics by improving communication and mentoring channels between the national and local frontline workers. In addition, tailoring training modules to the local scenarios will increase the relevance of the training to the Laos context.

## Data availability statement

The raw data supporting the conclusions of this article will be made available by the authors, without undue reservation.

## Ethics statement

The studies involving human participants were reviewed and approved by the University of Sydney Human Ethics Committee (2020/459 dated 15 September, 2020). Written informed consent for participation was not required for this study in accordance with the national legislation and the institutional requirements.

## Author contributions

SS, HT, and SF contributed to the study's conception, design, and questionnaire development. DM and VP contributed to the questionnaire translation and testing and managed logistics of the survey. HT managed the online questionnaire survey and database. SS and SF performed the data analysis. Funding was managed by SS, DM, VP, and HT. SS wrote the first draft of the manuscript. All authors contributed to manuscript revision, read, and approved the submitted version.
