# Supplementary material for: Field epidemiology capacity of the national veterinary services of Lao PDR: An online survey
Source: Front Vet Sci. 2023 Mar 21;10:1096554. doi: 10.3389/fvets.2023.1096554 (PMC10070838; doi:10.3389/fvets.2023.1096554)
Supplement: Supplementary file 1 [file Data_Sheet_1.PDF]

ການສຳຫຼວດຄວາມສາມາດທາງລະບາດວິທະຍາ ແລະປະເມີນຄວາມຕ້ອງການ
Epidemiology Capacity and Needs Assessment Survey

ໃນການສຳຫຼວດນີ້, ພວກເຮົາຈະໄດ້ສອບຖາມກ່ຽວກັບປະສົບການ ແລະ ຄຳຄິດເຫັນຂອງທ່ານກ່ຽວກັບຄວາມຕ້ອງການດ້ານທັກສະທາງລະບາດວິທະຍາໃນພື້ນທີ່. ການສຳຫຼວດນີ້ແມ່ນຈະໄດ້ປະຕິບັດຕາມແຕ່ລະພາກດັ່ງນີ້:
In this survey, we will ask you questions about your experiences and opinions about the need for various epidemiological skills in the field. The survey has the following sections:

- ການສືບສວນສອບສວນພະຍາດລະບາດ (Outbreak investigations)
- ການເຝົ້າລະວັງພະຍາດສັດ (Animal Disease Surveillance)
- ການຈັດການ ແລະ ການວິເຄາະຂໍ້ມູນ (Data Management and Analysis)
- ການສຳຫຼວດ ແລະ ການສຶກສາທາງລະບາດວິທະຍາ (Epidemiological surveys and studies)
- ສຸຂະພາບໜຶ່ງດຽວ (One health)
- ຄວາມເປັນຜູ້ນຳ ແລະ ການສື່ສານ (Leadership and communication)
- ການນຳໃຊ້ວິທີການດ້ານຄວາມປອດໄພ ແລະ ການຮັບປະກັນທາງຊີວະພາບ (Use of biosafety and biosecurity methods)
- ຂໍ້ມູນດ້ານປະຊາກອນ (Demographics)

ຂໍຂອບໃຈຜູ້ເຂົ້າຮ່ວມໃນການສຳຫຼວດ ຄວາມຄິດເຫັນຂອງທ່ານແມ່ນເປັນຂໍ້ມູນທີ່ສຳຄັນສຳຫຼັບການສຳຫຼວດນີ້.
Thank you for participating in our survey. Your feedback is important to us.

ການສືບສວນສອບສວນພະຍາດລະບາດ (Outbreak Investigation)

1. ທ່ານໄດ້ປະຕິບັດສິ່ງຕໍ່ໄປນີ້ເປັນປະຈຳຫຼາຍປານໃດໃນຊ່ວງປີທີ່ຜ່ານມາ? (How often have you done the followings during the last year?)

	ບໍ່ເຄີຍ (Never)	ດົນໆເທື່ອໜຶ່ງ (Rarely)	ປະມານເດືອນລະຄັ້ງ (About once a month)	ຫຼາຍກວ່າໜຶ່ງເທື່ອຕໍ່ເດືອນ (More than once a month)
ທ່ານໄດ້ປະຕິບັດກວດກາທາງຄີນິກເພື່ອການກວດຫາ ແລະ ການບົ່ງມະຕິພະຍາດບໍ່? (Conducted clinical examination for case detection and diagnosis?)	○	○	○	○
ທ່ານໄດ້ປະຕິບັດການຜ່າຊາກເພື່ອການກວດຫາ ແລະ ການບົ່ງມະຕິພະຍາດບໍ່? (Conducted postmortem examination for case detection and diagnosis?)	○	○	○	○

ທ່ານໄດ້ພັດທະນານິຍາມພະຍາດເພື່ອ ຈຳແນກສັດ ຫຼື ຟາມທີ່ເປັນພະຍາດ ແລະ ບໍ່ເປັນພະຍາດ? (Developed case definitions to classify animals or farms into cases and non-cases?	○	○	○	○
ທ່ານໄດ້ໃຊ້ນິຍາມພະຍາດເພື່ອ ຈຳແນກສັດ ຫຼື ຟາມທີ່ເປັນພະຍາດ ແລະ ບໍ່ເປັນພະຍາດບໍ່? (Applied case definitions toclassify animals or farms into cases and non-cases?)	○	○	○	○
ທ່ານໄດ້ພິສູດການເກີດການລະບາດພະຍາດບໍ່? (Verified that an outbreak is happening?)	○	○	○	○
ທ່ານໄດ້ປະຕິບັດການຄົ້ນຫາໄປທາງໜ້າ ແລະ ຍ້ອນກັບເພື່ອລະບຸການເກີດພະຍາດອື່ນບໍ່? (Conducted trace-forward and tracebackward searches to identify other cases?)	○	○	○	○
ທ່ານໄດ້ສ້າງແບບສອບຖາມໃນການສືບສວນສອບສວນ ພະຍາດລະບາດບໍ່? (Created an outbreak investigation questionnaire?)	○	○	○	○
ທ່ານໄດ້ເກັບຕົວຢ່າງບໍ່? (Collected samples?)	○	○	○	○
ທ່ານໄດ້ສ້າງແບບຟອມການສົ່ງຕົວຢ່າງບໍ່? (Created sample submission forms?)	○	○	○	○
ທ່ານໄດ້ໃຊ້ແບບຟອມການສົ່ງຕົວຢ່າງບໍ່? (Used sample submission forms?)	○	○	○	○
ທ່ານໄດ້ນຳສົ່ງຕົວຢ່າງໄປຫ້ອງວິເຄາະບໍ່? (Transported samples to the laboratory?)	○	○	○	○
ທ່ານໄດ້ຕີລາຄາຜົນຈາກຫ້ອງວິເຄາະບໍ່? (Interpreted laboratory results?)	○	○	○	○
ທ່ານໄດ້ວິເຄາະຂໍ້ມູນຈາກພະຍາດລະບາດ ຕາມພື້ນທີ່, ເວລາ ແລະ ກຸ່ມສັດ? (Analysed data from an outbreak by space, time and animal groups?)	○	○	○	○
ທ່ານໄດ້ນຳໃຊ້ຫຼັກການພື້ນຖານການຄວບຄຸມ ເພື່ອປ້ອງກັນການເກີດພະຍາດບໍ່? (Applied preliminary control strategies to contain the outbreak?)	○	○	○	○
ທ່ານໄດ້ຂຽນລາຍງານການເກີດພະຍາດບໍ່? (Produced an outbreak report?)	○	○	○	○

ຄຳຄິດເຫັນອື່ນໆ (Any comments): ______________________________

---

2. ທ່ານໄດ້ເປັນຜູ້ນຳ ຫຼື ຜູ້ເຂົ້າຮ່ວມໃນສືບສວນສອບສວນການເກີດພະຍາດ ຈັກຄັ້ງໃນຊ່ວງ 3 ປີຜ່ານມາ?

(How many times have you led or participated in an outbreak investigation in the past 3 years?)

- ◯ ບໍ່ເຄີຍ (Never)
- ◯ 1 - 2 ເທື່ອ (times)
- ◯ 3 - 6 ເທື່ອ (times)
- ◯ ຫຼາຍກວ່າ 6 ເທື່ອ (More than 6 times)

---

3. ຈາກທັກສະ ແລະ ປະສົບການຂອງຜູ້ເບິ່ງແຍງສັດໃນພື້ນທີ່ຮັບຜິດຊອບ ຂອງທ່ານ, ລະດັບຄວາມສໍາຄັນທີ່ທ່ານຄິດວ່າຕ້ອງການຝຶກອົບຮົມເພີ່ມຕື່ມໃນການດໍາເນີນການສືບສວນສອບສວນພະຍາດລະບາດມີໜ້ອຍຫຼາຍປານໃດ?
(Based on the skills and experiences of the animal workers in your LGA, what priority would you say there is for further training in conducting outbreak investigations at the moment?)

- ☐ ບໍ່ຕ້ອງການຝຶກອົບຮົມເພີ່ມ (No need for further training)
- ☐ ຕໍ່າ (Low priority)
- ☐ ປານກາງ (Moderate priority)
- ☐ ຫຼາຍ (High priority)
- ☐ ຫຼາຍທີ່ສຸດ (Very high priority)

ຄໍາຄິດເຫັນອື່ນໆ (Any comments):

____

ການເຝົ້າລະວັງທາງພະຍາດສັດ (Animal Disease Surveillance)

4. ທ່ານໄດ້ປະຕິບັດສິ່ງຕໍ່ໄປນີ້ເປັນປະຈຳຫຼາຍປານໃດໃນຊ່ວງປີທີ່ຜ່ານມາ? (How often have you done the followings during the last year?)

	ບໍ່ເຄີຍ Never	ດົນໆເທື່ອໜຶ່ງ Rarely	ປະມານເດືອນລະຄັ້ງ About once a month	ຫຼາຍກວ່າໜຶ່ງເທື່ອຕໍ່ເດືອນ More than once a month
ທ່ານໄດ້ລົງຢ້ຽມຢາມຟາມ ແລະ ໄດ້ສົນທະນາກັບຊາວກະສິກອນ ເພື່ອຊອກຫາພະຍາດທີ່ອາດເກີດຂື້ນໄດ້ບໍ່? (Visited farms and talked with farmers to identify possible cases?)	○	○	○	○
ທ່ານໄດ້ຕິດຕາມການລາຍງານຈາກແຫຼ່ງທີ່ເປັນທາງການຂໍ້ມູນບໍ່? (Followed up reports from informal sources?)	○	○	○	○
ທ່ານໄດ້ລາຍງານການເກີດພະຍາດ ຫຼື ກຸ່ມຂອງການເກີດພະຍາດໄປຫາໜ່ວຍງານທີ່ກ່ຽວຂ້ອງ? (Reported cases or clusters of cases to the appropriate authorities?)	○	○	○	○
ທ່ານໄດ້ເຮັດການສະຫຼຸບລາຍງານການເຝົ້າລະວັງບໍ່? (Produced a surveillance summary report?)	○	○	○	○
ທ່ານໄດ້ອອກແບບຮູບແບບການລະຫຼບລາຍງານການເຝົ້າລະວັງທີ່ສາມາດນຳໃຊ້ໄດ້ແຕ່ລະໄລຍະບໍ່? (Designed a surveillance summary report template that can be used periodically?)	○	○	○	○
ທ່ານໄດ້ອອກແບບລະບົບການເຝົ້າລະວັງບໍ່? (Designed a surveillance system?)	○	○	○	○
ທ່ານໄດ້ປະເມີນການເຮັດວຽກ ແລະ ອົງປະກອບຂອງການລາຍງານພະຍາດຂອງລະບົບການເຝົ້າລະວັງບໍ່? (Evaluated the operation and disease reporting components of surveillance system?)	○	○	○	○
ທ່ານໄດ້ບອກເຖິງຈຸດແຂງ, ຂໍ້ຈຳກັດ ແລະ ຊ່ອງວ່າງຂອງລະບົບການເຝົ້າລະວັງບໍ່? (Identified the strengths, limitations and gaps of a surveillance system?)	○	○	○	○

ຄຳຄິດເຫັນອື່ນໆ (Any comments):

5. ທ່ານໄດ້ເປັນຜູ້ນຳ ຫຼື ຜູ້ເຂົ້າຮ່ວມໃນກິດຈະກຳການເຝົ້າລະວັງການເກີດພະຍາດສັດຈັກຄັ້ງໃນຊ່ວງ 3 ປີຜ່ານມາ?
(How many times have you led or participated in an animal disease surveillance activity in the past 3 years?)

- ◯ ບໍ່ເຄີຍ (Never)
- ◯ 1 - 2 ເທື່ອ (times)
- ◯ 3 - 6 ເທື່ອ (times)
- ◯ ຫຼາຍກວ່າ 6 ເທື່ອ (More than 6 times)

6. ຈາກທັກສະ ແລະ ປະສົບການຂອງຜູ້ເບິ່ງແຍງສັດໃນພື້ນທີ່ຮັບຜິດຊອບ ຂອງທ່ານ, ລະດັບຄວາມສຳຄັນທີ່ທ່ານຄິດວ່າຕ້ອງການຝຶກອົບຮົມເພີ່ມຕື່ມໃນການເຝົ້າລະວັງພະຍາດສັດມີໜ້ອຍຫຼາຍປານໃດ?
(Based on the skills and experiences of the animal workers in your LGA, what priority would you say there is for further training in animal disease surveillance at the moment?)

- ☐ ບໍ່ຕ້ອງການຝຶກອົບຮົມເພີ່ມ (No need for further training)
- ☐ ຕ່ຳ (Low priority)
- ☐ ປານກາງ (Moderate priority)
- ☐ ຫຼາຍ (High priority)
- ☐ ຫຼາຍທີ່ສຸດ (Very high priority)

ຄຳຄິດເຫັນອື່ນໆ (Any comments):

ການຈັດການ ແລະ ການວິເຄາະຂໍ້ມູນ (Data Management and Analysis)

7. ທ່ານໄດ້ປະຕິບັດສິ່ງຕໍ່ໄປນີ້ເປັນປະຈໍາຫຼາຍປານໃດໃນຊ່ວງປີທີ່ຜ່ານມາ? (How often have you done the followings during the last year?)

	ບໍ່ເຄີຍ Never	ດົນໆເທື່ອໜຶ່ງ Rarely	ປະມານເດືອນລະຄັ້ງ About once a month	ຫຼາຍກວ່າໜຶ່ງເທື່ອຕໍ່ເດືອນ More than once a month
ທ່ານໄດ້ເຂົ້າຂໍ້ມູນການເຝົ້າລະວັງ ຫຼື ພະຍາດລະບາດໃນໂປຣແກຣມບໍ່? ຕົວຢ່າງ: MS excel (Entered surveillance or outbreak data into a spreadsheet programmes such as MS excel?)	○	○	○	○
ທ່ານໄດ້ກວດສອບຂໍ້ມູນການເຝົ້າລະວັງສໍາລັບການເຂົ້າຂໍ້ມູນທີ່ຜິດ ແລະ ການພິມຜິດບໍ່? (Verified surveillance data for data entry errors and typos?)	○	○	○	○
ທ່ານໄດ້ກະກຽມ ແລະ ຕີລາຄາເສັ້ນໂຄ້ງການລະບາດເພື່ອອະທິບາຍພະຍາດລະບາດບໍ່?(Prepared and interpreted an epidemic curve to describe the outbreak?)	○	○	○	○
ທ່ານໄດ້ນໍາສະເໜີຂໍ້ມູນການເຝົ້າລະວັງ ຫຼື ພະຍາດລະບາດໂດຍການນໍາໃຊ້ຕາຕະລາງ ແລະ ເສັ້ນສະແດງບໍ່? (Presented surveillance or outbreak data using tables and graphs?)	○	○	○	○
ທ່ານໄດ້ປະຕິບັດການທົດສອບທາງສະຖິຕິຂອງສົມມຸດຕິຖານບໍ່? (Conducted a statistical test of hypothesis?)	○	○	○	○
ທ່ານໄດ້ຄິດໄລ່ອັດຕາການຕິດເຊື້ອ ແລະ ອັດຕາການເກີດພະຍາດຈາກຂໍ້ມູນການເຝົ້າລະວັງບໍ່?(Calculated prevalence and incidence measures from surveillance data?)	○	○	○	○
ທ່ານໄດ້ປຽບທຽບອັດຕາການຕິດເຊື້ອ ແລະ ອັດຕາການເກີດພະຍາດລະຫວ່າງກຸ່ມເພື່ອລະບຸປັດໃຈສ່ຽງບໍ່? (Compared prevalence and incidence between groups to identify risk factors?)	○	○	○	○
ທ່ານໄດ້ລະບຸທ່າອ່ຽງ, ຮູບແບບ ແລະ ຄ່າຜິດປົກກະຕິໃນຂໍ້ມູນການເຝົ້າລະວັງບໍ່? (Identified trends, patterns, and outliers in surveillance data?	○	○	○	○
ທ່ານໄດ້ສ້າງແຜນທີ່ຈາກຂໍ້ມູນພະຍາດລະບາດ ຫຼື ການເຝົ້າລະວັງບໍ່? (Constructed maps from outbreak or surveillance data?)	○	○	○	○

ທ່ານໄດ້ລະບຸກຸ່ມຂອງພະຍາດທີ່ໜ້າສົງໄສບໍ່?(Identified suspected clusters of disease?)	◯	◯	◯	◯

ຄຳຄິດເຫັນອື່ນໆ (Any comments):

8. ທ່ານໄດ້ເປັນຜູ້ນຳ ຫຼື ຜູ້ເຂົ້າຮ່ວມໃນກິດຈະກຳການຈັດການ ແລະ ການວິເຄາະຂໍ້ມູນຈັກຄັ້ງໃນຊ່ວງ 3 ປີຜ່ານມາ?
(How many times have you led or participated in data management and analysis activities in the past 3 years?)

- ◯ ບໍ່ເຄີຍ (Never)
- ◯ 1 - 2 ເທື່ອ (times)
- ◯ 3 - 6 ເທື່ອ (times)
- ◯ ຫຼາຍກວ່າ 6 ເທື່ອ (More than 6 times)

9. ຈາກທັກສະ ແລະ ປະສົບການຂອງຜູ້ເບິ່ງແຍງສັດໃນພື້ນທີ່ຮັບຜິດຊອບຂອງທ່ານ, ລະດັບຄວາມສຳຄັນທີ່ທ່ານຄິດວ່າຕ້ອງການຝຶກອົບຮົມເພີ່ມຕື່ມໃນການຈັດການ ແລະ ການວິເຄາະຂໍ້ມູນມີໜ້ອຍຫຼາຍປານໃດ?
(Based on the skills and experiences of the animal workers in your LGA, what priority would you say there is for further training in data management and analysis at the moment?)

- ☐ ບໍ່ຕ້ອງການຝຶກອົບຮົມເພີ່ມ (No need for further training)
- ☐ ຕ່ຳ (Low priority)
- ☐ ປານກາງ (Moderate priority)
- ☐ ຫຼາຍ (High priority)
- ☐ ຫຼາຍທີ່ສຸດ (Very high priority)

ຄຳຄິດເຫັນອື່ນໆ (Any comments):

ການສຳຫຼວດ ແລະ ສຶກສາທາງລະບາດວິທະຍາ (Epidemiological surveys and studies)

10. ທ່ານໄດ້ປະຕິບັດສິ່ງຕໍ່ໄປນີ້ເປັນປະຈຳຫຼາຍປານໃດໃນຊ່ວງປີທີ່ຜ່ານມາ? (How often have you done the followings during the last year?)

	ບໍ່ເຄີຍ Never	ດົນໆເທື່ອໜຶ່ງ Rarely	ປະມານເດືອນລະຄັ້ງ About once a month	ຫຼາຍກວ່າໜຶ່ງເທື່ອຕໍ່ເດືອນ More than once a month
ທ່ານໄດ້ອອກແບບແບບສອບຖາມສຳລັບການເກັບຂໍ້ມູນບໍ່? (Designed a questionnaire for data collection?)	◯	◯	◯	◯
ທ່ານໄດ້ວາງແຜນ ແລະ/ຫຼື ປະຕິບັດການສຳຫຼວດບໍ່? (Planned and/or conducted a survey?)	◯	◯	◯	◯
ທ່ານໄດ້ວາງແຜນ ແລະ/ຫຼື ປະຕິບັດການສຶກສາແບບຕັດຂວາງບໍ່? (Planned and/or conducted a cross-sectional study?)	◯	◯	◯	◯
ທ່ານໄດ້ວາງແຜນ ແລະ/ຫຼື ປະຕິບັດການສຶກສາແບບຄວບຄຸມບໍ່? (Planned and/or conducted a case control study?)	◯	◯	◯	◯
ທ່ານໄດ້ວາງແຜນ ແລະ/ຫຼື ປະຕິບັດການສຶກສາຕາມກຸ່ມບໍ່? (Planned and/or conducted a cohort study?)	◯	◯	◯	◯

ທ່ານໄດ້ວາງແຜນ ແລະ/ຫຼື ປະຕິບັດ ການຄົ້ວຫາພະຍາດແບບມີສ່ວນຮ່ວມ ບໍ່? (Planned and/or conducted a participatory disease search?)	○	○	○	○
ທ່ານໄດ້ວາງແຜນ ແລະ/ຫຼື ປະຕິບັດ ການສ້າງແຜນທີ່ຕ່ອງໂສ້ບໍ່? (Planned and/or conducted a value chain mapping?)	○	○	○	○
ທ່ານໄດ້ວາງແຜນ ແລະ/ຫຼື ຄິດໄລກຳໄລລາຍໄດ້ສຳລັບຟາມບໍ່? (Planned and/or conducted gross margins for a farm?)	○	○	○	○
ທ່ານໄດ້ວາງແຜນ ແລະ/ຫຼື ປະຕິບັດ ການປະເມີນຄວາມສ່ຽງທາງດ້ານຄຸນນະພາບບໍ່? (Planned and/or conducted qualitative risk assessment?)	○	○	○	○
ທ່ານໄດ້ຄິດໄລ່ຂະໜາດຂອງຕົວຢ່າງບໍ່? (Calculated sample size?)	○	○	○	○
ທ່ານໄດ້ປະເມີນຜົນຂອງການບົ່ງມະຕິພະຍາດບໍ່? (Evaluated a diagnostic test?)	○	○	○	○
ທ່ານໄດ້ເຮັດການຄົ້ນຫາ ແລະ ວິເຄາະປຽບທຽບຜົນຈາກການຄົ້ນຄວ້າທີ່ໄດ້ຖືກຕີພິມບໍ່? (Conducted a literature review?)	○	○	○	○

ຄຳຄິດເຫັນອື່ນໆ (Any comments):

____

11. ທ່ານໄດ້ເປັນຜູ້ນຳ ຫຼື ຜູ້ເຂົ້າຮ່ວມໃນການປະຕິບັດການສຳຫຼວດ ແລະ ການສຶກສາທາງລະບາດວິທະຍາຈັກຄັ້ງໃນຊ່ວງ 3 ປີຜ່ານມາ?
(How many times have you led or participated in conducting epidemiological surveys or studies in the times past 3 years?)

- ○ ບໍ່ເຄີຍ (Never)
- ○ 1 - 2 ເທື່ອ (times)
- ○ 3 - 6 ເທື່ອ (times)
- ○ ຫຼາຍກວ່າ 6 ເທື່ອ (More than 6 times)

12. ຈາກທັກສະ ແລະ ປະສົບການຂອງຜູ້ເບິ່ງແຍງສັດໃນພື້ນທີ່ຮັບຜິດຊອບ ຂອງທ່ານ, ລະດັບຄວາມສຳຄັນທີ່ທ່ານຄິດວ່າຕ້ອງການຝຶກອົບຮົມເພີ່ມຕື່ມໃນການສຳຫຼວດ ແລະ ການສຶກສາທາງລະບາດວິທະຍາມີໜ້ອຍຫຼາຍປານໃດ?
(Based on the skills and experiences of the animal workers in your LGA, what priority would you say there is for further training in epidemiological surveys and studies at the moment?)

- ☐ ບໍ່ຕ້ອງການຝຶກອົບຮົມເພີ່ມ (No need for further training)
- ☐ ຕ່ຳ (Low priority)
- ☐ ປານກາງ (Moderate priority)
- ☐ ຫຼາຍ (High priority)
- ☐ ຫຼາຍທີ່ສຸດ (Very high priority)

ຄຳຄິດເຫັນອື່ນໆ (Any comments):

____

ສຸຂະພາບໜຶ່ງດຽວ

"ສຸຂະພາບໜຶ່ງດຽວ" ແມ່ນການຮ່ວມມືເຮັດວຽກຂອງຫຼາຍພາກສ່ວນ ແລະ ຂ້າມສາຂາວິຊາຕ່າງໆ ໂດຍມີເປົ້າໝາຍເພື່ອບັນລຸຜົນໄດ້ຮັບດ້ານສຸຂະພາບທີ່ດີທີ່ສຸດ. ການຮັບຮູ້ເຖິງການເຊື່ອມຕໍ່ລະຫວ່າງຄົນ, ສັດ, ພືດ ແລະ ສະພາບແວດລ້ອມຮ່ວມກັນ

One Health

'One Health' is a collaborative, multi-sectoral, and trans-disciplinary approach with the goal of achieving optimal health outcomes recognising the interconnection between people, animals, plants, and their shared environment.

13. ທ່ານໄດ້ປະຕິບັດສິ່ງຕໍ່ໄປນີ້ເປັນປະຈຳຫຼາຍປານໃດໃນຊ່ວງປີທີ່ຜ່ານມາ? (How often have you done the followings during the last year?)

	ບໍ່ເຄີຍ Never	ດົນໆເທື່ອໜຶ່ງ Rarely	ປະມານເດືອນລະຄັ້ງ About once a month	ຫຼາຍກວ່າໜຶ່ງເທື່ອຕໍ່ເດືອນ More than once a month
ທ່ານໄດ້ພັດທະນາໂປຣແກຣມການຄວບຄຸມສຳລັບພະຍາດຕິດແປດໃສ່ຄົນບໍ່? (Developed a control program for a zoonotic disease?)	○	○	○	○
ທ່ານໄດ້ຊ່ວຍ ຫຼື ນຳພາໃນການສືບສວນສອບສວນພະຍາດຕິດແປດໃສ່ຄົນບໍ່? (Assisted in or led the investigation of a zoonotic disease?)	○	○	○	○
ທ່ານໄດ້ຊ່ວຍ ຫຼື ນຳພາໃນການສືບສວນສອບສວນພະຍາດທີ່ບໍ່ຕິດແປດໃສ່ຄົນບໍ່?(Assisted in or led the investigation of a non-zoonotic human disease?)	○	○	○	○
ທ່ານໄດ້ເຂົ້າຮ່ວມໃນທີມງານທີ່ມີຄວາມຊ່ຽວຊານຈາກພາກສ່ວນລ້ຽງສັດ, ຄົນ ແລະ/ຫຼື ສິ່ງແວດລ້ອມບໍ່? (Participated in a team involving professionals from animals, human, and/or environmental sectors?)	○	○	○	○

ຄຳຄິດເຫັນອື່ນໆ (Any comments):

____

14. ທ່ານໄດ້ເປັນຜູ້ນຳ ຫຼື ຜູ້ເຂົ້າຮ່ວມໃນກິດຈະກຳສຸຂະພາບໜຶ່ງດຽວຈັກຄັ້ງໃນຊ່ວງ 3 ປີຜ່ານມາ?
(How many times have you led or participated in conducting epidemiological surveys or studies in the times past 3 years?)

- ○ ບໍ່ເຄີຍ (Never)
- ○ 1 - 2 ເທື່ອ (times)
- ○ 3 - 6 ເທື່ອ (times)
- ○ ຫຼາຍກວ່າ 6 ເທື່ອ (More than 6 times)

15. ຈາກທັກສະ ແລະ ປະສົບການຂອງຜູ້ເບິ່ງແຍງສັດໃນພື້ນທີ່ຮັບຜິດຊອບ ຂອງທ່ານ, ລະດັບຄວາມສຳຄັນທີ່ທ່ານຄິດວ່າຕ້ອງການຝຶກອົບຮົມເພີ່ມຕື່ມກ່ຽວກັບສຸຂະພາບໜຶ່ງດຽວມີໜ້ອຍຫຼາຍປານໃດ?
(Based on the skills and experiences of the animal workers in your LGA, what priority would you say there is for further training in One Health at the moment?)

- ☐ ບໍ່ຕ້ອງການຝຶກອົບຮົມເພີ່ມ (No need for further training)
- ☐ ຕ່ຳ (Low priority)
- ☐ ປານກາງ (Moderate priority)
- ☐ ຫຼາຍ (High priority)
- ☐ ຫຼາຍທີ່ສຸດ (Very high priority)

ຄຳຄິດເຫັນອື່ນໆ (Any comments):

____

ຄວາມເປັນຜູ້ນຳ ແລະ ການສື່ສານ (Leadership and Communication)

16. ທ່ານໄດ້ປະຕິບັດສິ່ງຕໍ່ໄປນີ້ເປັນປະຈຳຫຼາຍປານໃດໃນຊ່ວງປີທີ່ຜ່ານມາ? (How often have you done the followings during the last year?)

	ບໍ່ເຄີຍ<br>Never	ດົນໆເທື່ອໜຶ່ງ<br>Rarely	ປະມານເດືອນລະຄັ້ງ<br>About once a month	ຫຼາຍກວ່າໜຶ່ງເທື່ອຕໍ່ເດືອນ<br>More than once a month
ທ່ານໄດ້ພັດທະນາສື່ການຮຽນຮູ້ສໍາລັບຊາວກະສິກອນບໍ່? (Developed educational materials for farmers?)	○	○	○	○
ທ່ານໄດ້ພັດທະນາສື່ການຝຶກອົບຮົບສໍາລັບຜູ້ອື່ນໆທີ່ເຮັດວຽກດ້ານສຸຂະພາບສັດບໍ່?(Developed training materials for other animal health workers?)	○	○	○	○
ທ່ານໄດ້ກະກຽມການລາຍງານສໍາລັບໜ່ວຍງານສຸຂະພາບສັດບໍ່? (Prepared reports for animal health authorities?)	○	○	○	○
ທ່ານໄດ້ນໍາສະເໜີດ້ວຍວາຈາບໍ່? (Given an oral presentation?)	○	○	○	○
ທ່ານໄດ້ກະກຽມການນໍາສະເໜີຜ່ານສື່ບໍ່?(Prepared a media release?)	○	○	○	○
ທ່ານໄດ້ໃຫ້ສໍາພາດຜ່ານສື່ບໍ່? (Given a media interview?)	○	○	○	○
ທ່ານໄດ້ປະຕິບັດການສື່ສານທາງການຜ່ານອີເມວບໍ່? (Handled official communication by email?)	○	○	○	○
ທ່ານໄດ້ນໍາໃຊ້ການປະຊຸມຜ່ານວີດີໂອບໍ່? (Used video-conferencing tools?)	○	○	○	○
ທ່ານໄດ້ກະກຽມບົດຄັດຫຍໍ້ສໍາລັບສົ່ງເຂົ້າກອງປະຊຸມວິຊາການບໍ່? (Prepared an abstract for submission to a conference?)	○	○	○	○
ທ່ານໄດ້ກະກຽມບົດຄວາມສໍາລັບການເຜີຍແຜ່ໃນວາລະສານວິທະຍາສາດບໍ່? (Prepared a manuscript for publication in a scientific journal?)	○	○	○	○
ທ່ານໄດ້ເປັນຜູ້ເບິ່ງແຍງບໍ່? (Supervised staff?)	○	○	○	○
ທ່ານໄດ້ນໍາພາການສືບສວນສອບສວນທາງລະບາດວິທະຍາບໍ່? (Led an	○	○	○	○

epidemiological investigation?)

ທ່ານໄດ້ນຳພາທີມງານຕອບໂຕ້ ຫຼື ເປັນສູນກາງການຄວບຄຸມບໍ່? (Led a response team or a control centre?)	○	○	○	○
ທ່ານໄດ້ບໍລິຫານທີມງານບໍ່? (Managed a team?)	○	○	○	○
ທ່ານໄດ້ວາງແຜນໂຄງການທີ່ກ່ຽວຂ້ອງກັບການພັດທະນາ ຫຼື ການປະຕິບັດລະບົບການເຝົ້າລະວັງບໍ່? (Planned a project related to surveillance system development or implementation?)	○	○	○	○

ຄຳຄິດເຫັນອື່ນໆ (Any comments): ______________________________

17. ທ່ານໄດ້ເຂົ້າຮ່ວມໃນກິດຈະກຳການເປັນຜູ້ນຳ ແລະ ການສື່ສານຈັກຄັ້ງໃນຊ່ວງ 3 ປີຜ່ານມາ?
(How many times have you participated in leadership and communication activities in the past 3 years?)

- ○ ບໍ່ເຄີຍ (Never)
- ○ 1 - 2 ເທື່ອ (times)
- ○ 3 - 6 ເທື່ອ (times)
- ○ ຫຼາຍກວ່າ 6 ເທື່ອ (More than 6 times)

18. ຈາກທັກສະ ແລະ ປະສົບການຂອງຜູ້ເບິ່ງແຍງສັດໃນພື້ນທີ່ຮັບຜິດຊອບ ຂອງທ່ານ, ລະດັບຄວາມສຳຄັນທີ່ທ່ານຄິດວ່າຕ້ອງການຝຶກອົບຮົມເພີ່ມຕື່ມໃນຄວາມເປັນຜູ້ນຳ ແລະ ການສື່ສານມີໜ້ອຍຫຼາຍປານໃດ?
(Based on the skills and experiences of the animal workers in your LGA, what priority would you say there is for further training in leadership and communication at the moment?)

- ☐ ບໍ່ຕ້ອງການຝຶກອົບຮົມເພີ່ມ (No need for further training)
- ☐ ຕ່ຳ (Low priority)
- ☐ ປານກາງ (Moderate priority)
- ☐ ຫຼາຍ (High priority)
- ☐ ຫຼາຍທີ່ສຸດ (Very high priority)

ຄຳຄິດເຫັນອື່ນໆ (Any comments): ______________________________

ການນຳໃຊ້ວິທີການດ້ານຄວາມປອດໄພ ແລະ ການຮັບປະກັນທາງດ້ານຊີວະພາບ (Use of biosafety and biosecurity methods?)

19. ທ່ານໄດ້ນຳໃຊ້ອຸປະກອນປ້ອງກັນຕົນເອງ (PPE - ເຊັ່ນ: ຖົງມື, ເກີບໂບກ, ຜ້າອັດປາກ ແລະ ຊຸດກັນເປື້ອນ) ເປັນປະຈຳຫຼາຍປານໃດໃນການປະຕິບັດກັບສັດເຈັບຊ່ວງປີຜ່ານມາ?
(How often have you used personal protective equipment (PPE - such as gloves, boots, mask and gowns) for handling sick animals during the last year?)

	ບໍ່ເຄີຍ (Never)	ດົນໆເທື່ອໜຶ່ງ (Rarely)	ເປັນບາງເວລາ (Sometimes)	ຕະຫຼອດເວລາ (Always)
ທ່ານໄດ້ໃຊ້ຖົງມືບໍ່? (Used gloves?)	○	○	○	○
ທ່ານໄດ້ໃຊ້ເກີບໂບກບໍ່? (Used gumboots?)	○	○	○	○
ທ່ານໄດ້ໃຊ້ຜ້າອັດປາກບໍ່? (Used a surgical mask?)	○	○	○	○
ທ່ານໄດ້ໃຊ້ຊຸດຄຸມ/ຊຸດກັນເປື້ອນບໍ່? (Used overalls/gown?)	○	○	○	○
ທ່ານໄດ້ໃຊ້ແວ່ນຕາ/ໜ້າກາກປ້ອງກັນບໍ່?(Used safety goggles/face shield?)	○	○	○	○
ທ່ານໄດ້ໃຊ້ຜ້າອັດປິດປາກ P2 ຫຼື N95 ບໍ່? (Used P2 or N95 respirators?)	○	○	○	○
ທ່ານໄດ້ກຳຈັດສິ່ງເສດທີ່ຕິດເຊື້ອບໍ່? (Disposed of infectious materials?)	○	○	○	○

ຄຳຄິດເຫັນອື່ນໆ (Any comments): ______________________________

20. ທ່ານໄດ້ລົງຢ້ຽມຢາມຟາມຈັກຄັ້ງໃນປີຜ່ານມາ?
(How many times have you visited a farm in the past year?)

____

21. ເມື່ອທ່ານລົງຢ້ຽມຢາມຟາມ, ທ່ານໄດ້ໃຊ້ວິທີການດ້ານຄວາມປອດໄພທາງດ້ານຊີວະພາບຕໍ່ໄປນີ້ເລື້ອຍໆບໍ່ໃນຊ່ວງປີຜ່ານມາ?
When visiting farms, how often have you used the following biosecurity methods during the last year?

	ບໍ່ເຄີຍ (Never)	ດົນໆເທື່ອໜຶ່ງ (Rarely)	ເປັນບາງເວລາ (Sometimes)	ຕະຫຼອດເວລາ (Always)
ທ່ານໄດ້ອານາໄມເກີບໂບກກ່ອນ ແລະ ຫຼັງຢ້ຽມຢາມຟາມບໍ່? (Cleaned boots before and after visiting a farm?)	○	○	○	○
ທ່ານໄດ້ລ້າງມືດ້ວຍສະບູ ແລະ ນ້ຳ ກ່ອນ ແລະ ຫຼັງຢ້ຽມຢາມຟາມບໍ່? (Washed hands with soap and water before and after visiting a farm?)	○	○	○	○
ທ່ານໄດ້ອານາໄມພາຫະນະກ່ອນ ແລະ ຫຼັງຢ້ຽມຢາມຟາມບໍ່? (Cleaned your vehicle before and after visiting a farm?)	○	○	○	○
ທ່ານໄດ້ຂ້າເຊື້ອອຸປະກອນຂອງທ່ານ ກ່ອນ ແລະ ຫຼັງຢ້ຽມຢາມຟາມບໍ່? (Disinfected your equipment before and after visiting a farm?)	○	○	○	○

ຄຳຄິດເຫັນອື່ນໆ (Any comments):

____

22. ຈາກທັກສະ ແລະ ປະສົບການຂອງຜູ້ເບິ່ງແຍງສັດໃນພື້ນທີ່ຮັບຜິດຊອບ ຂອງທ່ານ, ລະດັບຄວາມສຳຄັນທີ່ທ່ານຄິດວ່າຕ້ອງການຝຶກອົບຮົມເພີ່ມຕື່ມໃນດ້ານຄວາມປອດໄພ ແລະ ການຮັບປະກັນທາງຊີວະພາບມີໜ້ອຍຫຼາຍປານໃດ?
(Based on the skills and experiences of the animal workers in your LGA, what priority would you say there is for further training in biosafety and biosecurity methods at the moment?)

- ☐ ບໍ່ຕ້ອງການຝຶກອົບຮົມເພີ່ມ (No need for further training)
- ☐ ຕ່ຳ (Low priority)
- ☐ ປານກາງ (Moderate priority)
- ☐ ຫຼາຍ (High priority)
- ☐ ຫຼາຍທີ່ສຸດ (Very high priority)

ຄຳຄິດເຫັນອື່ນໆ (Any comments):

____

23. ອາຍຸຜູ້ໃຫ້ສໍາພາດ? (What is your age?)

☐ ຕໍ່າກວ່າ 18 (Under 18)
☐ 18-24
☐ 25-34
☐ 35-44
☐ 45-54
☐ 55-64
☐ 65+

24. ເພດ? (What is your gender?)

☐ ຍິງ (Female)
☐ ຊາຍ (Male)
☐ ອື່ນໆ (Other)

ອື່ນໆ (Other?)

____

25. ພື້ນທີ່ເຮັດວຽກຂອງທ່ານແມ່ນຫຍັງ? (What is your work area?)

ແຂວງ (Province)

____

ເມືອງ (District)

____

ກຸ່ມບ້ານ (Village Tract)

____

ບ້ານ (Village)

____

ລະຫັດບ້ານ (VillagePcode)

____

26. ໜ້າທີ່ປະຈຸບັນຂອງທ່ານ? (What is your current position?)

☐ ພະນັກງານສັດຕະວະແພດ (DLF veterinary staff)
☐ ສັດຕະວະແພດແຂວງ (PAFO veterinary staff)
☐ ສັດຕະວະແພດເມືອງ (DAFO veterinary staff)
☐ ສັດຕະວະແພດບ້ານ ຫຼື ແພດບ້ານ (Para-veterinarian or blue-cross worker Public Health officer)
☐ ອື່ນໆ (ລະບຸ) (Other (Specify))

ອື່ນໆ (ລະບຸ) (Other (Specify)):

____

27. ການສຶກສາສູງສຸດໃນສາຍວິທະຍາສາດສັດຕະວະແພດຂອງທ່ານ?
What is your highest level of education in veterinary science?

☐ ໃບຢັ້ງຢືນ (Diploma)
☐ ປະລິນຍາຕີ (Bachelors)
☐ ປະລິນຍາໂທ (Masters)
☐ ປະລິນຍາເອກ (Doctorate)
☐ ອື່ນໆ (ກະລຸນາລະບຸ) (Other(please specify))

ອື່ນໆ (ກະລຸນາລະບຸ) (Other (please specify))

28. ທ່ານຈົບການສຶກສາປີໃດ? (In which year did you complete that education?)

29. ທ່ານປະຕິບັດໜ້າທີ່ປະຈຸບັນຂອງທ່ານດົນປານໃດແລ້ວ (ປີ)?
About how long have you been in your current position (years)?

30. ທ່ານເຄີຍໄດ້ຮັບການຝຶກອົບຮົມທາງດ້ານລະບາດວິທະຍາອື່ນໆນອກຈາກປະລິນຍາສັດຕະວະແພດບໍ່? (ເລືອກໄດ້ຫຼາຍກວ່າໜຶ່ງຄໍາຕອບ) (Have you received any formal epidemiology training other than in your veterinary degree (select all that apply)?)	☐ ບໍ່ (No)<br>☐ ເຂົ້າຮ່ວມປະຊຸມວິຊາການດ້ານລະບາດວິທະຍາ (Attended epidemiology workshops)<br>☐ ລົງທະບຽນ ຫຼື ສໍາເລັດການຝຶກອົບຮົມດ້ານລະບາດວິທະຍາພາກສະ ໜາມ (Enrolled in or completed a field epidemiology training programme)<br>☐ ລົງທະບຽນ ຫຼື ສໍາເລັດການສຶກສາສູງກວ່າປະລິນຍາຕີດ້ານລະບາດວິທະຍາ (Enrolled in or completed a postgraduate qualification in epidemiology)<br>☐ ອື່ນໆ (ກະລຸນາລະບຸ) ຫຼື ລະບຸລະດັບຂອງການຝຶກອົບຮົມດ້ານລະບາດວິທະຍາທີ່ໄດ້ຮັບ (Other (please specify) or Specify the level of epidemiology training undertaken)

ອື່ນໆ (ກະລຸນາລະບຸ) ຫຼື ລະບຸລະດັບຂອງການຝຶກອົບຮົມດ້ານລະບາດວິທະຍາທີ່ໄດ້ຮັບ (Other (please specify) or Specify the level of epidemiology training undertaken)

ຂອບໃຈຫຼາຍໆສໍາລັບການຕອບແບບສໍາຫຼວດ! ຂໍ້ມູນຂອງທ່ານແມ່ນມີຄວາມສໍາຄັນສໍາລັບໂຄງການຂອງພວກເຮົາ.
Thank you for completing the survey! Your input will be valuable for our project.
